# Mitochondrial-Protective Effects of R-Phenibut after Experimental Traumatic Brain Injury

**DOI:** 10.1155/2020/9364598

**Published:** 2020-11-21

**Authors:** Einars Kupats, Gundega Stelfa, Baiba Zvejniece, Solveiga Grinberga, Edijs Vavers, Marina Makrecka-Kuka, Baiba Svalbe, Liga Zvejniece, Maija Dambrova

**Affiliations:** ^1^Latvian Institute of Organic Synthesis, Riga, Latvia; ^2^Department of Neurology and Neurosurgery, Riga Stradins University, Riga, Latvia; ^3^Latvia University of Life Sciences and Technologies, Jelgava, Latvia; ^4^Department of Pharmaceutical Chemistry, Riga Stradins University, Riga, Latvia

## Abstract

Altered neuronal Ca^2+^ homeostasis and mitochondrial dysfunction play a central role in the pathogenesis of traumatic brain injury (TBI). R-Phenibut ((3R)-phenyl-4-aminobutyric acid) is an antagonist of the *α*_2_*δ* subunit of voltage-dependent calcium channels (VDCC) and an agonist of gamma-aminobutyric acid B (GABA-B) receptors. The aim of this study was to evaluate the potential therapeutic effects of R-phenibut following the lateral fluid percussion injury (latFPI) model of TBI in mice and the impact of R- and S-phenibut on mitochondrial functionality *in vitro*. By determining the bioavailability of R-phenibut in the mouse brain tissue and plasma, we found that R-phenibut (50 mg/kg) reached the brain tissue 15 min after intraperitoneal (i.p.) and peroral (p.o.) injections. The maximal concentration of R-phenibut in the brain tissues was 0.6 *μ*g/g and 0.2 *μ*g/g tissue after i.p. and p.o. administration, respectively. Male Swiss-Webster mice received i.p. injections of R-phenibut at doses of 10 or 50 mg/kg 2 h after TBI and then once daily for 7 days. R-Phenibut treatment at the dose of 50 mg/kg significantly ameliorated functional deficits after TBI on postinjury days 1, 4, and 7. Seven days after TBI, the number of Nissl-stained dark neurons (N-DNs) and interleukin-1beta (IL-1*β*) expression in the cerebral neocortex in the area of cortical impact were reduced. Moreover, the addition of R- and S-phenibut at a concentration of 0.5 *μ*g/ml inhibited calcium-induced mitochondrial swelling in the brain homogenate and prevented anoxia-reoxygenation-induced increases in mitochondrial H_2_O_2_ production and the H_2_O_2_/O ratio. Taken together, these results suggest that R-phenibut could serve as a neuroprotective agent and promising drug candidate for treating TBI.

## 1. Introduction

Traumatic brain injury (TBI) is a leading cause of mortality and disability among trauma-related injuries [1]. TBI can result in temporary, long-term, and even life-long physical, cognitive, and behavioural problems [2, 3]. Therefore, there is an increased need for effective pharmacological approaches for treating patients with TBI. Phenibut, a nootropic prescription drug with anxiolytic activity, is used in clinical practice in Eastern European countries for the treatment of anxiety, tics, stuttering, insomnia, dizziness, and alcohol abstinence [4, 5]. R-Phenibut ((3R)-phenyl-4-aminobutyric acid), which is one of the optical isomers of phenibut, binds to gamma-aminobutyric acid B (GABA-B) receptors and the *α*_2_*δ* subunit of voltage-dependent calcium channels (VDCC), while S-phenibut binds only to the *α*_2_*δ* subunit of VDCC [6–8]. Our previous studies have shown that R-phenibut treatment significantly decreased the brain infarct size and increased brain-derived neurotrophic factor and vascular endothelial growth factor gene expression in damaged brain tissue in an experimental stroke model [9]. The similarity of the pathogenic mechanisms of TBI and cerebral ischaemia indicate that therapeutic strategies that are successful in treating one may also be beneficial in treating the other [10].

Treatment options for TBI are limited due to its complex pathogenesis and the heterogeneity of its presentation, which includes haematomas, contusions, hypoxia, and vascular, axonal, and other types of central nervous system injuries [11, 12]. Among the processes that impact TBI, the generation of reactive oxygen species (ROS) by mitochondria occurs within the first minutes after TBI and thus leads to the disruption of calcium ion (Ca^2+^) homeostasis, which is the “final common pathway” for toxic cellular degradation [13, 14]. Maintaining regional neuronal Ca^2+^ homeostasis and mitochondrial function is crucial to prevent secondary neuronal injury [15, 16]. Thus, mitochondrial-targeted drugs and drugs acting on specific intracellular Ca^2+^ signalling pathways or subcellular components show promise as therapeutic interventions for TBI [17, 18]. In fact, upregulation of the neuronal calcium channel *α*_2_*δ* subunit modulates the activation of mitochondrial Ca^2+^ buffering in pathological conditions [19]. There is also evidence that GABA-B receptor agonists provide neuroprotection against N-methyl-D-aspartate-induced neurotoxicity mediated by the mitochondrial permeability transition pore [20]. Since both isomers of phenibut bind to the *α*_2_*δ* subunit of VDCC and only R-phenibut binds to the GABA-B receptor, these both isomers could be used to specify the possible molecular mechanisms of phenibut in different experimental models.

This is the first investigation of the potential therapeutic effects of R-phenibut following TBI in mice. In addition, to evaluate possible molecular mechanisms underlying the actions of R-phenibut against anoxia-reoxygenation-induced mitochondrial damage, the effects on mitochondrial functionality were evaluated in an *in vitro* model of anoxia-reoxygenation and compared for R- and S-phenibut.

## 2. Materials and Methods

### 2.1. Animals and Treatment

Forty-eight Swiss-Webster male mice (25-40 g; Laboratory Animal Centre, University of Tartu, Tartu, Estonia) were used in a lateral fluid percussion injury (latFPI) model of TBI [21, 22]. Additionally, 6 Swiss-Webster male mice were used for the preparation of brain homogenate and the isolation of brain mitochondria for *in vitro* assays. Forty-two ICR male mice (Laboratory Animal Breeding Facility, Riga Stradins University, Latvia) were used in a pharmacokinetic study. All animals were housed under standard conditions (21-23°C, 12 h light-dark cycle) with unlimited access to standard food (Lactamin AB, Mjölby, Sweden) and water in an individually ventilated cage housing system (Allentown Inc., Allentown, New Jersey, USA). Each cage contained bedding consisting of Eco-Pure ™ Shavings wood chips (Datesand, Cheshire, UK), nesting material, and wooden blocks from TAPVEI (TAPVEI, Paekna, Estonia). For enrichment, a transparent tinted (red) nontoxic durable polycarbonate safe harbour mouse retreat (AnimaLab, Poznan, Poland) was used. The mice were housed with up to 5 mice per standard cage (38 × 19 × 13 cm). All studies involving animals were reported in accordance with the ARRIVE guidelines [23, 24]. The experimental procedures were performed in accordance with the guidelines reported in the EU Directive 2010/63/EU and in accordance with local laws and policies; all procedures were approved by the Latvian Animal Protection Ethical Committee of Food and Veterinary Service in Riga, Latvia.

The dose of R-phenibut was selected based on the previous studies, where pharmacological efficacy was observed in dose-range between 10 and 50 mg/kg, while R-phenibut at doses higher than 100 mg/kg showed sedative and coordination inhibitory effects [6, 8, 9]. Mice were randomly assigned to four experimental groups: sham-operated mice, saline-treated latFPI mice, and latFPI mice that received R-phenibut (JSC Olainfarm, Olaine, Latvia) at a dose of 10 mg/kg or 50 mg/kg. Six mice were excluded because of a dural breach that occurred during surgery (4 mice from the sham-operated, 1 mouse from the control, and 1 mouse from the R-phenibut 50 mg/kg groups), and four mice died immediately after latFPI and were excluded from the study (3 mice from the control and 1 mouse from the R-phenibut 50 mg/kg groups). The final number of included animals per group was as follows: sham-operated mice (*n* = 8), saline-treated latFPI mice (control group, *n* = 8), and latFPI mice that received R-phenibut at a dose of 10 mg/kg (*n* = 12) or 50 mg/kg (*n* = 10). R-Phenibut and saline were initially administered intraperitoneally (i.p.) 2 h after injury and then once daily for an additional 7 days for a total treatment period of 1 week. During the treatment period, the animals were weighed at 0, 1, 2, 4, and 7 days after latFPI between 9:00 and 10:00 am. To avoid the influence of subjective factors on the rating process, all experimental procedures were performed in a blinded fashion.

### 2.2. Determination of R-Phenibut in the Plasma and Brain Tissue after p.o. and i.p. Administration

The concentrations of R-phenibut in the brain tissue extracts and plasma were measured by ultraperformance liquid chromatography-tandem mass spectrometry (UPLC/MS/MS). To determine the concentration of R-phenibut in the plasma and brain, mice received an i.p. and p.o. R-phenibut at a dose of 50 mg/kg 15 and 30 min and 1, 2, 4, 6, and 24 h (*n* = 3 in each time point) before the plasma and brain tissue collection. The blood and brain samples were prepared as described previously [25]. The chromatographic separation was performed using an ACQUITY UPLC system (Waters, USA) on an ACQUITY UPLC BEH Shield RP18 (1.7 *μ*m, 2.1 × 50 mm) (Waters) with a gradient elution from 5 to 98% acetonitrile in 0.1% formic acid aqueous solution at a flow rate of 0.15 ml/min. The analyte was ionized by electrospray ionization in positive ion mode on a Quattro Micro triple quadrupole mass spectrometer (Waters). The mass spectrometer was set up as follows: capillary voltage of 3.3 kV; source and desolvation temperatures of 120 and 400°C, respectively. Cone voltage was 20 V, and collision energy was 18 eV. R-Phenibut analysis was performed in the MRM mode. Precursor to production transition was *m*/*zm*/*z* 180.0→116.1. Data acquisition and processing were performed using the MassLynx V4.1 and QuanLynxV4.1 software (Waters).

### 2.3. Lateral Fluid Percussion Injury-Induced Brain Trauma

To induce TBI, the latFPI model was generated as previously described [21, 22] with slight modifications. Mice were anaesthetized with 4% isoflurane contained in a mixture of oxygen and nitrous oxide (70 : 30, AGA, Riga, Latvia), and anaesthesia with 2% isoflurane (Chemical Point, Deisenhofen, Germany) was maintained during the surgical procedures using a face mask. The depth of anaesthesia was monitored by a toe pinch using tweezers. Before trauma induction, mice received subcutaneous (s.c.) administration of tramadol (KRKA, Novo Mesto, Slovenia) (10 mg/kg). Eye cream was applied to prevent the eyes from drying out. A midline longitudinal scalp incision was made, and the skull was exposed. A craniectomy that was centred at 2 mm posterior to bregma and 2 mm right of midline was performed using a 3 mm outer-diameter trephine. Any animal noted to have a dural breach was euthanized and excluded from the study. A plastic cap was attached over the craniotomy using dental cement (Fulldent, Switzerland), and a moderate severity (1.5 ± 0.2 atm) brain injury was induced with a commercially available fluid percussion device (AmScien Instruments, Richmond, USA). Immediately after the injury, apnoea was noted, and when spontaneous breathing returned, anaesthesia was resumed. The cement and cap were removed, and the skin was sutured using resorbable sutures (6-0, silk). The animal was placed in a separate cage to allow full recovery from anaesthesia. Sham-injured animals were subjected to an identical procedure as the latFPI animals except for the induction of trauma.

### 2.4. Neurological Severity Score (NSS)

The neurobehavioural status of mice was obtained by the NSS using the method described previously [26]. The animals were trained on the NSS beams and equipment prior to the baseline measurements. The general neurological state of mice was evaluated at baseline (day before latfTBI) and 1, 4, and 7 days postinjury before the next dose of R-phenibut or saline administration. The NSS consisted of 9 individual clinical parameters, including motor function, alertness, and physiological behaviour tasks. The mice were assessed for the following items: presence of paresis; impairment of seeking behaviour; absence of perceptible startle reflex; inability to get down from a rectangle platform (34 × 27 cm); inability to walk on 3, 2, and 1 cm wide beams; and inability to balance on a vertical beam of 7 mm width and horizontal round stick of 5 mm diameter for 10 sec. If a mouse showed impairment on one of these items, a value of 1 was added to its NSS score. Thus, higher scores on the NSS indicate greater neurological impairment.

### 2.5. Tissue Preparation for Histological Analysis

The animals used for histological analysis were randomly selected from each group. Seven days after TBI, the mice were anaesthetized using i.p. administration of ketamine (200 mg/kg) and xylazine (15 mg/kg). The depth of anaesthesia was monitored by a toe pinch using tweezers. Animals were transcardially perfused at a rate of 3 ml/minutes with 0.01 M phosphate-buffered saline (PBS, pH = 7.4) for 5 minutes until the blood was completely removed from the tissue. Perfusion was then performed with 4% paraformaldehyde (PFA) fixative solution for 5-7 minutes until stiffening of the mouse body occurred. After perfusion, the brains were carefully dissected and postfixed in 4% PFA overnight at 4°C. The brains were cryoprotected with a 10-20-30% sucrose-PBS gradient for 72 hours. Coronal sections of the brain (20 *μ*m) were made using a Leica CM1850 cryostat (Leica Biosystems, Buffalo Grove, IL, United States) and mounted on Superfrost Plus microscope slides (Thermo Scientific, Waltham, MA, United States).

### 2.6. Cresyl Violet (Nissl) Staining and Interleukin-1beta (IL-1*β*) Immunofluorescence Staining

Nissl and IL-1*β* staining techniques were used to evaluate neuronal cell damage. Nissl-stained dark neurons (N-DNs) indicated the typical morphological change in injured neurons following TBI [27, 28]. The number of N-DNs and cells expressing IL-1*β* in the cerebral neocortex in the cortical impact area were determined at day 7 after latFPI. For Nissl staining, coronal frozen sections (20 *μ*m) of the mouse brain were used. The sections were incubated in graded ethanol solutions (96% ethanol for 3 minutes and 70% ethanol for 3 minutes). After washing with distilled water for 3 minutes, the sections were stained with 0.01% cresyl violet acetate (ACROS organics) solution for 14 minutes. The sections were then washed with distilled water for 3 minutes and dehydrated in ethanol. The stained sections were coverslipped using DPX mounting medium (Sigma-Aldrich, St. Louis, MO, United States).

For IL-1*β* staining, the sections were washed once with PBS containing 0.2% Tween 20 for 5 minutes (on a rotary shaker at 250 rpm). The antigen retrieval procedure was performed with 0.05 M Na citrate (pH = 6.0) containing 0.05% Tween 20 for 30 minutes at 85°C. The sections were then washed with PBS (0.2% Tween 20) 3 times for 5 minutes each. Protein blocking was performed using 5% BSA solution, and the sections were incubated for 1 hour at room temperature. The sections were washed with PBS (0.2% Tween 20) 3 times for 5 minutes each. The slices were incubated with primary antibody against anti-IL-1*β* (1 : 1000; Abcam, Cat# ab9722) for 16 h at +4°C. The antibody was diluted in PBS containing 3% BSA and 0.3% Triton™ X-100. After incubation with the primary antibody, the sections were washed with PBS (0.2% Tween 20) 4 times for 5 minutes each. The sections were subsequently incubated for 1 h at room temperature with goat anti-rabbit IgG H&L (Alexa Fluor® 488, 1 : 200; Abcam, Cat# ab150077) diluted in PBS containing 5% BSA. The sections were washed with PBS (0.2% Tween 20) 4 times for 5 minutes each. The stained sections were mounted using Fluoromount™ aqueous mounting medium (Sigma-Aldrich, St. Louis, MO, United States, Cat# F4680) and finally coverslipped. Images were obtained with a Nikon Eclipse TE300 microscope (Nikon Instruments, Tokyo, Japan).

N-DNs were defined as hyperbasophilic neurons with a shrunken morphology. The number of N-DNs per field of vision was calculated in three randomly selected sections at the epicentre of the injury. The number of N-DNs and cells expressing IL-1*β* per field of vision were calculated using ImageJ software at 10-fold magnification for N-DNs and at 4-fold magnification for IL-1*β*. For analysis of expression of IL-1*β*, eight-bit images were generated from the pictures and were cropped to contain the regions of interest. Images for IL-1*β* staining were thresholded to select a specific signal over the background, and the stained area for each region was calculated and used for statistical analysis. Three individual measurements were performed for each sample. The schematic illustration of the brain region was created using BioRender software (https://biorender.com).

### 2.7. Mitochondrial Respiration and H_2_O_2_ Production Measurements

To evaluate mitochondrial functionality, mouse brain homogenate or isolated brain mitochondria were prepared. Briefly, brain tissues were homogenized 1 : 20 (*w*/*v*) in a medium containing 320 mM sucrose, 10 mM Tris, and 1 mM EDTA (pH 7.4). The homogenate was centrifuged at 1000 g for 10 min, and the supernatant was centrifuged at 6200 g for 10 min. The mitochondrial pellet obtained was washed once and resuspended in the isolation medium. Mitochondrial respiration and H_2_O_2_ production measurements were performed at 37°C using Oxygraph-2k (O2k; Oroboros Instruments, Austria) with O2k-Fluo-Modules in MiR05Cr (110 mM sucrose, 60 1mM K-lactobionate, 0.5 mM EGTA, 3 mM MgCl_2_, 20 mM taurine, 10 mM KH_2_PO_4_, 20 mM HEPES, pH 7.1, 0.1% BSA essentially fatty acid free, and creatine 20 mM). H_2_O_2_ flux (ROS flux) was measured simultaneously with respirometry in the O2k-fluorometer using the H_2_O_2_-sensitive probe Ampliflu™ Red (AmR) [29, 30]. 10 *μ*M AmR, 1 U/ml horse radish peroxidase (HRP), and 5 U/ml superoxide dismutase (SOD) were added to the chamber. H_2_O_2_ detection is based on the conversion of AmR into the fluorescent resorufin. Calibrations were performed with H_2_O_2_ added at 0.1 *μ*M step. H_2_O_2_ flux was corrected for background (AmR slope before addition of sample). H_2_O_2_/O flux ratio (%) was calculated as H_2_O_2_ flux/(0.5 O_2_ flux).

### 2.8. In Vitro Anoxia-Reoxygenation Model

Mitochondrial functionality after anoxia-reoxygenation was determined in mouse brain tissue homogenate prepared as described previously [31]. To induce anoxia maximal respiration rate, the sample was stimulated by the addition of substrates, pyruvate + malate (5 + 2 mM), succinate (10 mM), and ADP (5 mM), and preparation was left to consume all O_2_ in the respiratory chamber (within 10-20 min), thereby entering into an anoxic state [32]. 15 minutes after anoxia, the vehicle or R-phenibut (0.5 *μ*g/ml) was added to the chamber and O_2_ was reintroduced to the chamber by opening the chamber to achieve reoxygenation. After 8 minutes of reoxygenation, the chamber was closed and O_2_ flux was monitored for additional 2 minutes. At the end of the experiment, antimycin A (2.5 *μ*M) was added to determine residual oxygen consumption (ROX).

### 2.9. Substrate-Uncoupler-Inhibitor Titration (SUIT) Protocol

To determine the effect of R-phenibut on mitochondrial electron transfer system functionality, mitochondria were isolated from mouse brain as described previously, and mitochondrial respiration and H_2_O_2_ production measurements were performed in the presence or absence of R-phenibut at 0.5 *μ*g/ml concentration [30]. In addition, effects of S-phenibut (0.5 *μ*g/ml) were tested to determine whether the effects of R-phenibut in mitochondria involve the GABA-B receptor or the *α*_2_*δ* subunit of VDCC. Pyruvate and malate (5 mM and 2 mM, respectively) were used to determine N-pathway complex I (CI) linked LEAK (L) respiration. ADP was added at 5 mM concentration to determine oxidative phosphorylation-dependent respiration (OXPHOS state, P). Then, glutamate (10 mM) was added as an additional substrate for N-pathway. Succinate (10 mM, complex II (CII) substrate) was added to reconstitute convergent NS-pathway CI&II-linked respiration. Titrations with the uncoupler CCCP (0.5–1 *μ*M steps) were performed to determine the electron transfer system (ETS) capacity. Rotenone (0.5 *μ*M, inhibitor of complex I) was added to determine the CII-linked OXPHOS capacity. Then, antimycin A (2.5 *μ*M, inhibitor of complex III) was added to evaluate residual (non-mitochondrial) oxygen consumption (ROX). Oxygen fluxes were compared after correction for ROX.

### 2.10. Ca^2+^-Induced Mitochondrial Swelling Measurement

Swelling of isolated brain mitochondria was assessed by measuring changes in absorbance at 540 nm as described previously with slight modifications [33–35]. Mitochondria (0.125 mg/ml) were preincubated with R- or S- phenibut at a concentration of 0.5 *μ*g/ml for 15 min in a buffer containing 120 mM KCl, 10 mM Tris, 5 mM KH_2_PO_4_ pH 7.4, and pyruvate (5 mM), malate (2 mM), and ADP (5 mM) as substrates. R- and S-enantiomers of phenibut were used to determine whether the effects of R-phenibut on Ca^2+^-induced mitochondrial swelling involve the GABA-B receptor or the *α*_2_*δ* subunit of VDCC. Swelling was induced by the addition of 200 *μ*M CaCl_2_, and changes in absorbance were monitored for 10 min. All experiments were performed at 37°C.

### 2.11. Statistical Analysis

All results are expressed as the mean ± S.E.M or S.D. (for mitochondrial studies). Health outcomes, animal behaviour, and Ca^2+^-induced mitochondrial swelling were analysed using two-way repeated-measures analysis of variance (ANOVA). Dunnett's post hoc test was performed when appropriate. The histological data and mitochondrial functionality were evaluated by one-way ANOVA. Whenever the analysis of variance indicated a significant difference, further multiple comparisons were made using Tukey's multiple comparison test as the post hoc test. *p* values less than 0.05 were considered to be significant. The statistical calculations were performed using the GraphPad Prism software package (GraphPad Software, Inc., La Jolla, California, USA).

The sample size calculations for latFPI-induced brain trauma were based on the effects of R-phenibut in our previous experiments. For example, it was calculated that R-phenibut demonstrates a medium effect in the ET-1-induced middle cerebral artery occlusion model [9] and a large effect in the formalin-induced paw-licking test [6]. Through a power calculation (using G-power software) for a two-way ANOVA test (repeated measures), four-group comparison, four measurements per group (0, 1, 4, and 7 days after TBI) with *α* = 0.05, a power of 80%, and a standardized effect size Cohen's *f* = 0.5, a total sample size of 8 mice per group was deemed sufficient. Since TBI-induced brain trauma can result in death of some animals, our sample size of *n* = 12 would allow identifying smaller differences, with the same statistical power, for the same significance level.

## 3. Results

### 3.1. R-Phenibut Crosses the Blood-Brain Barrier

**Figure 1:**
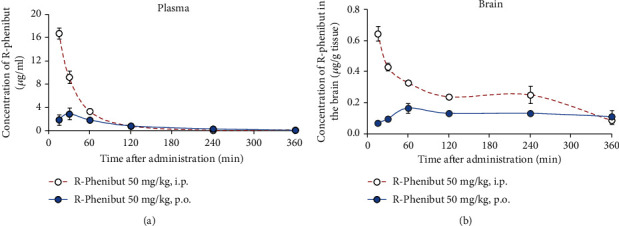
The concentration of R-phenibut in the mouse plasma and brain tissue after a single administration. Mice received an i.p. and p.o. injection of R-phenibut at a dose of 50 mg/kg. The amount of compound in the plasma (a) and brain tissue extracts (b) was measured 15 and 30 min and 1, 2, 4, and 6 h after R-phenibut administration (*n* = 3). Values are represented as the mean ± S.E.M..

As shown in Figure 1(a), R-phenibut in plasma could be detected 15 min after a single i.p. and p.o. injection. The maximal concentrations of R-phenibut in the plasma were observed 15 min after the i.p. injection and 30 min after the p.o. administration (Figure 1(a)). The maximal concentration of R-phenibut in the plasma after the i.p. injection was 16.8 *μ*g/ml; at the same time, the maximal concentration of R-phenibut in the plasma after the p.o. injection was 24 *μ*g/ml (see Figure 1(a)). R-Phenibut in the plasma was not detected 24 h after both the i.p. and p.o. injections. R-Phenibut in the brain tissue extracts was detected already 15 min after a single i.p. and p.o. injection (Figure 1(b)). The maximal concentrations of R-phenibut in the brain tissues were 0.64 *μ*g/g and 0.17 *μ*g/g tissue after the i.p. and p.o. injections, respectively (Figure 1(a)). The maximal concentrations of R-phenibut in the brain tissues were observed 15 min after i.p. injection and 60 till 240 min after p.o. administration. 24 h after both the i.p. and p.o. injections, R-phenibut in the brain tissues was 0.02 *μ*g/g and 0.012 *μ*g/g, respectively.

### 3.2. Health Outcome Monitoring after latFPI

**Figure 2:**
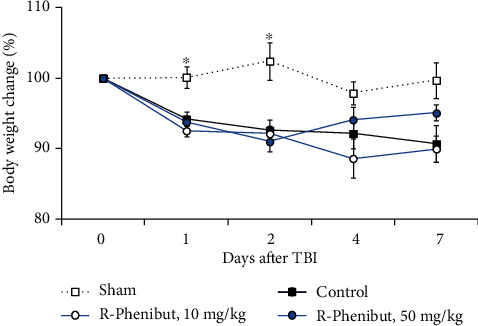
Body weight changes of the sham-operated, control, and R-phenibut treatment groups. Mice were weighed before and 1, 2, 4, and 7 days after latFPI. Data are expressed as the percentage change in body weight relative to the initial body weight of each animal (%). Data are shown as the mean ± S.E.M. (*n* = 8 − 12). ^∗^Indicates a significant difference compared to the sham-operated group (two-way repeated-measures ANOVA followed by Dunnett's multiple comparison test; ^∗^*P* < 0.05).

The body weight of the sham group animals was not decreased at 1, 2, 4, and 7 days after TBI. A two-way repeated-measures ANOVA showed a significant interaction between time and treatment (*F*_(12, 118)_ = 4.6, *p* < 0.0001) and main effects of time (*F*_(1.4, 42.7)_ = 25.7, *p* < 0.0001) and treatment (*F*_(3, 34)_ = 6.7, *p* = 0.0011). The control group animals lost significantly more weight after TBI than the sham-operated group animals (*p* < 0.05). Treatment with R-phenibut at both doses had no effect on weight loss compared to weight loss in the control group (Figure 2).

### 3.3. R-Phenibut Treatment Improved Neurological Status after TBI

**Figure 3:**
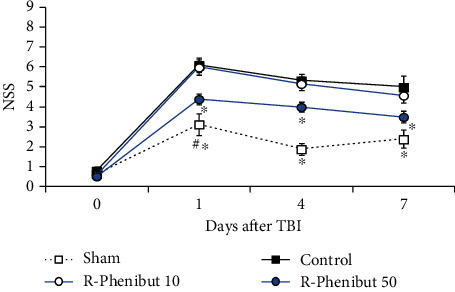
Effects of R-phenibut on the neurological severity score (NSS) after TBI. R-Phenibut and saline were initially administered i.p. 2 h after injury and then once daily for an additional 7 days for a total treatment period of 1 week. Data are shown as the mean ± S.E.M. (*n* = 8 − 12). ^∗^Indicates a significant difference compared to the control group; ^#^indicates a significant difference compared to the sham-operated group (two-way repeated-measures ANOVA followed by Dunnett's multiple comparison test; ^∗^*P* < 0.05; ^#^*P* < 0.01).

TBI induced significant functional deficits in control mice compared with sham-operated mice (*p* < 0.0001). The average NSS in the control group was 6.1 ± 0.4, 5.3 ± 0.3, and 5.0 ± 0.6 on postinjury days 1, 4, and 7, respectively. The average NSS score between baseline value and the first day postcraniotomy in the sham-operated group was significantly higher (*p* < 0.01). There was a significant time × treatmentinteraction observed between groups (two-way repeated-measures ANOVA: (*F*_(9, 102)_ = 5.7, *p* < 0.0001) for time × treatmentinteraction; (*F*_(3, 34)_ = 22.2, *p* < 0.0001) for treatment; (*F*_(2.7, 92)_ = 161.8, *p* < 0.0001) for time; Figure 3). R-Phenibut treatment at a dose of 50 mg/kg significantly ameliorated functional deficits by 28%, 25%, and 30% after TBI on postinjury days 1, 4, and 7, respectively (*p* < 0.05; Figure 3).

### 3.4. R-Phenibut Treatment Reduced Early Neuronal Cell Death and Neuroinflammation in the Brain Cortex after TBI

**Figure 4:**
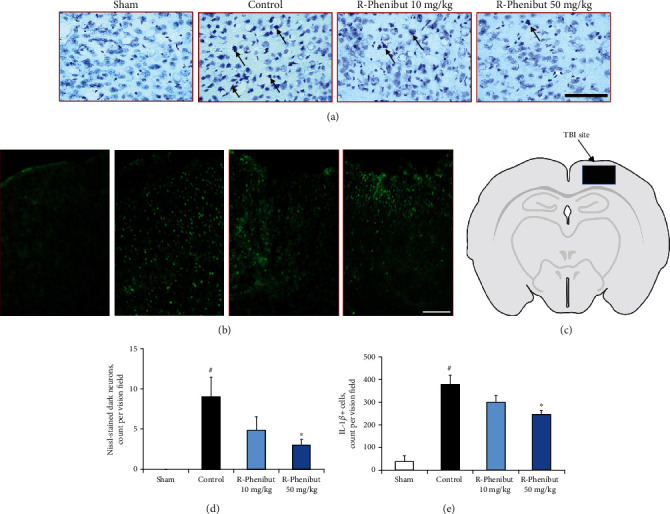
Cresyl violet (Nissl) and IL-1*β* immunofluorescence staining 7 days post-TBI. (a) Cresyl violet-stained sections of the mouse neocortex ipsilateral to the injury site. R-Phenibut treatment at doses of 10 mg/kg and 50 mg/kg reduced the number of N-DNs. Scalebar = 100*μ*m. (b) IL-1*β* expression based on immunofluorescence staining in the mouse neocortex ipsilateral to the injury site. R-Phenibut treatment at doses of 10 mg/kg and 50 mg/kg reduced the number of IL-1*β*-positive cells. Scalebar = 250*μ*m. (c) Schematic illustration of the brain region indicated in the filled area, which was selected for the quantitative analysis of cell injury. (d) Quantitative assessment of N-DNs in the ipsilateral cortex at postinjury day 7. Data are expressed as the mean ± S.E.M. (*n* = 7 for the R-phenibut 50 mg/kg group and n = 6 for the sham, control, and R-phenibut 10 mg/kg groups). (e) Quantitative assessment of IL-1*β*-positive cells in the ipsilateral cortex at postinjury day 7. Data are expressed as the mean ± S.E.M. (*n* = 4 for the control group and *n* = 3 for the sham, R-phenibut 10 mg/kg, and 50 mg/kg groups). ^#^Indicates a significant difference compared to the sham-operated group; ^∗^indicates a significant difference compared to the control group (one-way ANOVA followed by Tukey's multiple comparison test; ^∗^*P* < 0.05).

To assess histopathological changes in the ipsilateral brain site, N-DNs and cells expressing IL-1*β* (Figure 4) were quantified in the sham-operated, control, and R-phenibut treatment groups 7 days after TBI. N-DNs and IL-1*β*-expressing cells were found in the ipsilateral hemisphere of control group animals (Figures 4(a) and 4(b)). Histological analysis showed that R-phenibut treatment at a dose of 50 mg/kg significantly reduced the number of N-DNs and cells expressing IL-1*β* in the neocortex after TBI (*p* < 0.05). Significant differences were found in the N-DNs and IL-1*β*-positive cell numbers in the ipsilateral cortex around the lesion site between the control group (9.1 ± 6.4/per field of vision for N-DNs and 379 ± 82/per field of vision for IL-1*β*-expressing cells) and the R-phenibut treatment group at a dose of 50 mg/kg (3.0 ± 1.9/per field of vision for N-DNs and 246 ± 31/per field of vision for IL-1*β*-expressing cells; *p* < 0.05; Figures 4(d) and 4(e)). There was no statistically significant difference between the control group and the R-phenibut treatment group at the dose of 10 mg/kg. No N-DNs were observed in the sham-operated mice.

### 3.5. R-Phenibut Protects Brain Mitochondria against Anoxia-Reoxygenation Damage

**Figure 5:**
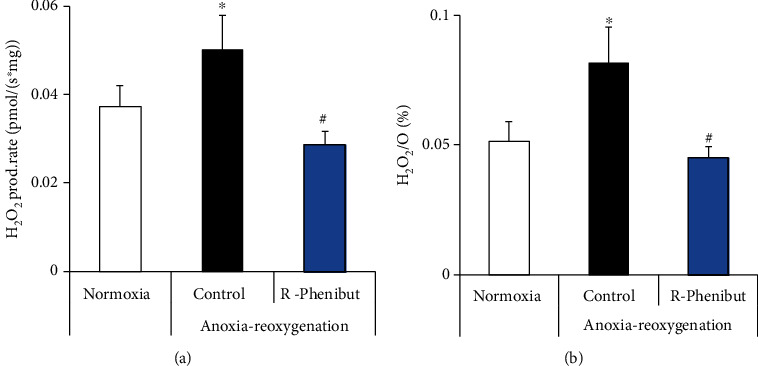
The effects of R-phenibut (0.5 *μ*g/ml) on ROS production in an *invitro* anoxia-reoxygenation model. After anoxia-reoxygenation, the H_2_O_2_ production rate (a) and H_2_O_2_/O ratio (b) were significantly decreased in the R-phenibut group. The results are presented as the mean ± S.D. of 6 independent replicates. ^∗^Indicates a significant difference compared to normoxia; ^#^indicates a significant difference compared to the anoxia-reoxygenation control group (one-way ANOVA followed by Tukey's multiple comparison test; ^∗^*P* < 0.05).

To determine whether R-phenibut-induced neuroprotection could be a result of the preservation of mitochondrial functionality, ROS production and the mitochondrial respiration rate were assessed after anoxia-reoxygenation *in vitro*. To better mimic the conditions observed *in vivo*, R-phenibut at the concentration of 0.5 *μ*g/ml was added to the chamber immediately before reoxygenation. Anoxia-reoxygenation induced 33% and 59% increases in the H_2_O_2_ production rate and the H_2_O_2_/O ratio, respectively (Figure 5). R-Phenibut treatment significantly decreased the anoxia-reoxygenation-induced increase in the H_2_O_2_ production rate and the H_2_O_2_/O ratio (*p* < 0.05).

### 3.6. R-Phenibut Reduces ROS Production and Attenuates Ca^2+^-Induced Mitochondrial Swelling

**Figure 6:**
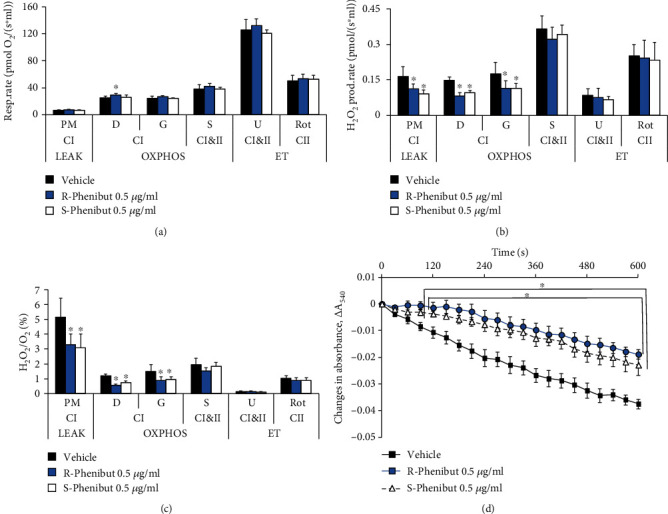
The effects of R-phenibut and S-phenibut (0.5 *μ*g/ml) on mitochondrial functionality and Ca^2+^-induced swelling in isolated mouse brain mitochondria. R-Phenibut and S-phenibut did not affect the mitochondrial respiration rate (a) but significantly decreased the H_2_O_2_ production rate (b) and H_2_O_2_/O ratio (c). The results are presented as the mean ± S.D. of 5 independent measurements. P: pyruvate; M: malate; D: ADP; G: glutamate; S: succinate; U: uncoupler; Rot: rotenone; CI: complex I; CII: complex II; LEAK: substrate metabolism-dependent state; OXPHOS: oxidative phosphorylation-dependent state; ET: electron transfer capacity state. ^∗^Indicates a significant difference compared to the control group (one-way ANOVA followed by Tukey's multiple comparison test, ^∗^*P* < 0.05). Both R-phenibut and S-phenibut at a concentration of 0.5 *μ*g/ml significantly attenuated Ca^2+^-induced swelling (d). The results are presented as the mean ± S.D. of 7 independent replicates. ^∗^Indicates a significant difference compared to the control group (two-way repeated-measures ANOVA followed by Dunnett's multiple comparison test; ^∗^*P* < 0.05).

To determine whether the protective effect of R-phenibut is related to its direct action on mitochondria, measurements of mitochondrial respiration, ROS production, and Ca^2+^-induced swelling were performed in isolated mouse brain mitochondria in the presence or absence of the compounds. As seen in Figure 6, R-phenibut and S-phenibut at 0.5 *μ*g/ml did not induce any changes in the mitochondrial respiration rate (Figure 6(a)), while H_2_O_2_ production and the H_2_O_2_/O ratio (Figures 6(b) and 6(c)) were significantly decreased by 31-53% in the LEAK and OXPHOS states. These results show that R-phenibut and S-phenibut reduce ROS production without affecting the mitochondrial electron transfer system capacities, indicating the improvement of mitochondrial coupling. In addition, both R- and S-phenibut attenuated calcium-induced brain mitochondrial swelling (two-way repeated-measures ANOVA: main effect of treatment (*F*_(40, 360)_ = 4.576, *p* < 0.0001), time (*F*_(2.611, 46.99)_ = 104.5, *p* < 0.0001), and interaction between treatment and time (*F*_(40, 360)_ = 4.576, *p* < 0.0001); Figure 6(d)).

Thus, the phenibut treatment-induced protection of mitochondria against anoxia-reoxygenation could be due to a reduction in ROS production and the modulation of Ca^2+^ signalling.

## 4. Discussion

In the current study, we examined the effects of R-phenibut treatment on brain trauma induced by latFPI. For the first time, we showed that R-phenibut could be detected in the mouse brain 15 min after a single p.o. or i.p. injection and found in brain extracts even 24 h after the administration. The present study confirms that R-phenibut, which is an antagonist of the *α*_2_*δ* subunit of VDCC and an agonist of GABA-B receptors, improves sensorimotor functional outcomes and significantly ameliorates brain damage and neuronal death in the acute phase after TBI via mechanisms related to Ca^2+^ homeostasis and oxidative stress.

The binding characteristics of R-phenibut were previously investigated using radiolabeled gabapentin that was the first ligand shown to bind to the *α*_2_*δ*_1_ and *α*_2_*δ*_2_ subunits with high affinity (K_d_ = 59 and 153 nM, respectively), while at the same time demonstrating no binding activity to the *α*_2_*δ*_3_ and *α*_2_*δ*_4_ subunits [36, 37]. The pathologies associated with gene disruption of *α*_2_*δ*_1_ protein include neuropathic pain and cardiac dysfunction, while in case of *α*_2_*δ*_2_ protein, the pathologies are related to epilepsy and cerebellar ataxia [38]. We showed previously that pharmacological activity of R-phenibut is associated with neuropathic pain rather than epilepsy [6]; thus, we could speculate that the effects of R-phenibut are *α*_2_*δ*_1_ protein binding-related.

The *α*_2_*δ* subunits of VDCC are widely expressed by excitatory neurons in the cerebral cortex, hippocampus, and other brain regions [39, 40]. Furthermore, the *α*_2_*δ* subunits of VDCC have been shown to be involved in processes that are not directly linked to calcium channel function, such as synaptogenesis [41]. Other studies have reported that the administration of VDCC ligands in rodent models of TBI reduced cell death and improved cognitive function [40]. Similar to phenibut, ligands of the *α*_2_*δ* subunit of VDCC, such as pregabalin, at a high dose of 60 mg/kg reduce neuronal loss and improve functional outcomes 24 h after trauma in experimental models of TBI [41, 42]. Moreover, pregabalin at a dose of 30 mg/kg has been shown to improve functional recovery and to demonstrate anti-inflammatory and antiapoptotic effects in a rat model of spinal cord injury [43, 44].

Cytoskeletal protein loss results in altered neuronal morphology after TBI [45, 46]. N-DNs represent a typical pathomorphological change in injured neurons after TBI, showing abnormal basophilia and shrinkage [27, 28]. N-DNs appear in the neocortex immediately after TBI and can be observed even two weeks postinjury [27, 47]. In addition, IL-1 is a major driver of the secondary neuronal injury cascade after TBI [48]. It is involved in the recruitment of other types of immune cells, neuronal apoptosis, and blood-brain barrier disruption after TBI [49–51]. Furthermore, IL-1*β* antagonism was shown to be neuroprotective in clinical trials and in rodent models of TBI [52–54]. The present study shows that treatment with R-phenibut at a dose of 50 mg/kg significantly reduced the number of N-DNs and significantly reduced IL-1*β* expression in the neocortex after TBI. The histopathological findings of the current study revealed that R-phenibut could attenuate neuronal damage, inflammation, and degeneration.

For the first time, we showed that R-phenibut limits mitochondrial dysfunction in the brain induced by anoxia-reoxygenation. Compared with other types of cells, neurons are endowed with less robust antioxidant defence systems [55]. As mitochondrial dysfunction has been shown to be involved in TBI, perturbations in energy metabolism are likely to contribute to the pathogenesis of TBI [56, 57]. In TBI, oxidative cell damage is caused by an imbalance between the production and accumulation of ROS, in which mitochondria are the major intracellular source of ROS. Accordingly, there is accumulating evidence that antioxidant agents and membrane lipid peroxidation inhibitors, such as tirilazad, U-78517F and U-83836E, are effective in treating preclinical models of TBI [17]. Mitochondrial-targeted drugs, such as mitoquinone and thymoquinone-containing antioxidants, have been shown to decrease neurological deficits and *β*-amyloid-induced neurotoxicity after TBI [58, 59]. Meanwhile, the inhibition of ROS production has been shown to inhibit secretion of IL-1*β* [60].

Notably, the immunosuppressant drug cyclosporine A, which is an IL-1*β* receptor antagonist, has been shown to decrease pathological changes in the brain after TBI by blocking the mitochondrial permeability transition pore [61]. Our results indicate that R-phenibut treatment improves mitochondrial tolerance and thus protects brain energetics against anoxia-reoxygenation damage by reducing ROS production. R-Phenibut treatment reduces ROS production without affecting the mitochondrial electron transfer system capacities, indicating the improvement of mitochondrial coupling. Another study has demonstrated that phenibut has neuroprotective effects *in vitro* but does not possess antioxidant potential [62]. Perfilova et al. recently showed that phenibut can limit heart and brain mitochondrial damage in rats exposed to stress [63].

To determine the molecular mechanisms underlying the actions of R-phenibut against anoxia-reoxygenation-induced mitochondrial damage, the activity of the R- and S-enantiomers of racemic phenibut was compared. We found that both R-phenibut and S-phenibut reduced mitochondrial ROS production and inhibited Ca^2+^-induced mitochondrial swelling. This suggests that the protective effects of R-phenibut in mitochondria do not involve the GABA-B receptor (in contrast to R-phenibut, S-phenibut does not bind to the GABA-B receptor) and might be mediated by the *α*_2_*δ*_1_ subunit of VDCC. It was shown previously that increased intracellular Ca^2+^, as a result of increased activity of *α*_2_*δ*_1_, could be rapidly taken up by mitochondria and subsequently released into the cytoplasm avoiding Ca^2+^ accumulation and maintaining intracellular Ca^2+^ signalling [19]. This could explain why, in the presence of R- and S-phenibut, reduced Ca^2+^-induced mitochondrial swelling was observed. Both R-phenibut and S-phenibut demonstrate mitochondrial-protective properties against anoxia-reoxygenation and Ca^2+^-induced stress. Since there is no evidence of *α*_2_*δ* localization in the mitochondrial membrane, it is possible that compounds could alter Ca^2+^ signalling pathways and protect mitochondria by targeting mitochondrial-specific or mitochondrial-endoplasmatic reticulum-associated Ca^2+^ transporters.

Our study has several limitations. One of the limitations of this study is that the level of ROS in mouse brain after treatment of R-phenibut following TBI was not measured. Another limitation is the increase in the NSS score between the baseline value and the first day postcraniotomy in the sham-operated group. The increase of the NSS score in sham-operated mice was reported previously and can be related to the distinct injury caused by craniotomy procedures [64]. Similar to other studies, the NSS score of injured mice showed maximum deficits on postinjury day 1 and remained elevated at 1, 2, 4, and 7 days after latFPI [64, 65]. A potential limitation of this study is that only male mice were used in experiments.

## 5. Conclusions

In conclusion, R-phenibut treatment reduces TBI-induced neuronal death and improves functional recovery, suggesting its therapeutic potential. The present study suggests that the neuroprotective properties of phenibut may be mediated by its effects on mitochondrial calcium influx and ROS generation.

## Data Availability

The data used to support the findings of this study are available from the corresponding author upon request.
